# CAR-T overdrive: harnessing inosine for metabolic rewiring and stemness induction

**DOI:** 10.1038/s41392-024-01818-z

**Published:** 2024-05-07

**Authors:** Martí Farrera-Sal, Michael Schmueck-Henneresse

**Affiliations:** Berlin Institute of Health (BIH) at Charité—Universitätsmedizin Berlin, BIH Center for Regenerative Therapies (BCRT), Experimental Immunotherapy, Augustenburger Platz 1, Berlin, Germany

**Keywords:** Immunotherapy, Tumour immunology, Cancer

In a recent *Cancer Cell* publication, Klysz et al. investigated the impact of inosine (INO), a catabolic product of adenosine (Ado), on Chimeric Antigen Receptor (CAR) T-cell function, stemness, and exhaustion. They provided insights into T-cell exhaustion metabolic pathways and proposed mechanisms for overcoming it through metabolic reprogramming.^1^

CAR-T-cell therapy stands as a ground-breaking advancement in cancer treatment. Patients’ T cells are genetically engineered to express a chimeric receptor targeting tumour antigens, followed by expansion ex vivo before reinfusion. Engineered CAR-T cells hold promise against refractory B-cell malignancies but face challenges such as tumour relapse due to antigen loss and limited efficacy against solid tumours due to target identification, impaired CAR-T-cell tumour trafficking, and the highly immunosuppressive tumour microenvironment (TME), leading to T-cell exhaustion. T-cell exhaustion manifests as decreased effector function, inhibitory receptors expression, impaired cytokine production, reduced proliferation, and lower metabolic activity. In addition, the phenomenon of tonic signalling, characterised by autonomous CAR activation in the absence of tumour antigen stimulation, exacerbates cellular exhaustion. Therefore, understanding exhaustion dynamics and promoting CAR-T-cell stemness becomes paramount for enhancing CAR-T-cell therapy efficacy.

**Fig. 1:**
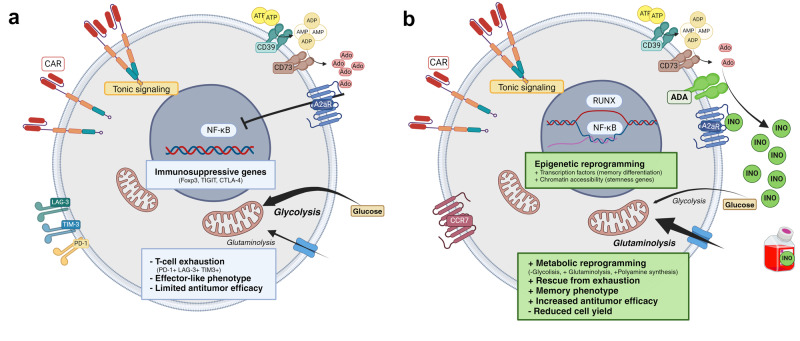
Inosine rescues exhausted CAR-T-cell products. **a** Traditional CAR-T manufacturing can lead to tonic signalling, resulting in exhausted phenotypes characterised by the expression of PD-1, TIM-3, LAG-3, and metabolic enzymes such as CD39 and CD73. These enzymes degrade ATP into adenosine (Ado), an immunosuppressive metabolite sensed by the A2aR receptor. **b** Overexpression of adenosine deaminase (ADA, shown in green) catabolizes Ado into inosine (INO). Alternatively, direct supplementation of INO can also rescue the exhaustion phenotype by inhibiting A2aR signalling, inducing epigenetic and metabolic reprogramming, and promoting an active memory-like phenotype of the CAR-T-cell product. Figure made in Biorender.com

The authors used an in vitro model of CAR-T-cell exhaustion induced by tonic signalling. CAR-T cells targeting the GD2 antigen exhibited heightened tonic signalling and an abundance of CD39 and CD73 enzymes, which metabolise ATP to Ado, during CAR-T-cell production. CD39^+^CD73^+^ CAR-T cells, representing highly dysfunctional exhausted T-cell subsets, displayed immunosuppressive properties via Ado production and signalling through the Ado receptor (A2aR, as depicted in Fig. 1a). To mitigate exhaustion, the authors aimed to reduce Ado levels by knocking out CD39, CD73, or A2aR, but observed only modest effects. However, increasing Ado catalysis into inosine (INO) by overexpressing the adenosine deaminase enzyme (ADA, depicted in green in Fig. 1b) or directly supplementing INO during CAR-T production resulted in a higher frequency of stem cell memory subsets and reduced progenitor and terminally exhausted CAR-T cells (Fig. 1b). This data points towards a direct INO effect, elucidating why reducing Ado levels (via CD39-KO or CD73-KO) showed modest effects compared to the increase of INO. Remarkably, INO can serve as an alternative energy source to glucose for T cells and is metabolically more stable (half-life ~15 h) than Ado (half-life <10 s).^2^ INO supplementation increased glutaminolysis and polyamine synthesis, and induced the production of ornithine decarboxylase, spermine, and EIF5a. Elevated polyamine levels have been associated with EIF5A hypusination involved in metabolic adaptation, aging, development, and immune cell differentiation.^3^ Supporting these findings, knockdown of EIF5A or hypusination inhibition decreased central memory-like CAR-T-cells frequency, implicating polyamine metabolism in INO-dependent induction of stemness.

GD2-CAR-T cells reprogrammed by overexpressing ADA enzyme (increasing INO levels) positively enriched gene sets resembling those of CD19-CARs (no tonic signalling) as well as CD19-CAR-T cells associated with long-term remissions. Furthermore, INO addition rescued already exhausted T cells, augmenting stemness and effector function even at later stages of CAR-T-cell manufacturing, which represents a significant advancement in CAR-T-cell production. The data confirm an active role of INO in T cells, remodelling chromatin and enhancing gene programs associated with stemness, increased adenosine resistance and improved effector function. The memory phenotype of CAR-T products has been linked to improved outcomes in B-cell malignancies, particularly those treated with CD19-CARs exhibiting residual tonic signalling. However, it remains unclear if this association holds true for solid tumours.^4^ The authors utilised both exhaustion-prone (GD2-CAR) and non-exhaustion-prone (CD19-CAR) cells during manufacturing, and INO-reprogramming substantially enhanced both antitumor effectiveness, improving potency and persistence in leukaemia and solid tumour murine models.

While the study offers valuable insights from in vitro and in vivo experiments, the effectiveness of inosine reprogramming in patient-derived CAR-T-cell production remains to be validated clinically. Considering the reduced cell expansion of the INO-reprogrammed CAR-T cells, future research should aim to confirm the results particularly for patients with low T-cell counts due to extensive pre-treatment. Clarifying this is vital and may necessitate re-evaluating dosing protocols in clinical trials.^5^ Forthcoming studies should investigate potential long-term or unintended off-target effects of metabolic reprogramming. Finally, the CAR construct design impacts metabolism, phenotype, persistence, and potential toxicity. Notably, the study focused on 41BB CARs, which may limit the generalisability of findings to other CAR constructs. Further investigations should explore the applicability of inosine reprogramming in CARs with different costimulatory domains, particularly in solid tumour settings.

Given the considerable metabolic adaptability of T cells, Klysz et al. findings open new avenues for enhancing CAR-T-cell products through metabolic reprogramming. While existing strategies target adenosine production or signalling in the TME, the findings suggest a shift towards approaches aimed at catabolizing adenosine to inosine and triggering polyamine synthesis, which may prove more efficacious. Emerging evidence emphasizes the crucial connection between T-cell metabolism, function, and persistence, urging exploration of metabolic adjustments to improve CAR-T-cell therapies. Potential strategies include optimising culture conditions, supplementing with cytokines and amino acids, and pharmacologically modulating metabolic pathways. Recent advancements in abbreviated CAR-T culture techniques raise questions regarding the need or the sustained efficacy of inosine supplementation. Investigating its effectiveness within modified culture conditions is essential. However, INO’s capacity to augment stemness and effector function in exhausted T cells hints at its potential applicability across diverse culture settings.

We anticipate a growing emphasis on metabolic reprogramming to tailor CAR-T products for specific tumour types, as suggested by Klysz et al. Future investigations exploring advanced culture conditions and INO supplementation could yield valuable insights for refining CAR-T-cell therapies and improving clinical outcomes. Finally, considering the profound influence of INO supplementation on CAR-T-cell function and stemness, it is tempting to speculate on the potential correlation between serum inosine levels, or metabolites that trigger polyamine synthesis, and efficacy in CAR-T trials. This could provide valuable insights into patient stratification and optimisation of treatment approaches.

In conclusion, this study offers a novel approach to mitigate T-cell exhaustion and enhance therapeutic efficacy through metabolic reprogramming. These findings pave the way for innovative advancements in tailoring CAR-T products, emphasising the critical role of metabolic optimisation in improving clinical outcomes across diverse tumour types.
